# Joint association of cigarette smoking and PM_2.5_ with COPD among urban and rural adults in regional China

**DOI:** 10.1186/s12890-021-01465-y

**Published:** 2021-03-15

**Authors:** Jian Su, Qing Ye, Dandan Zhang, Jinyi Zhou, Ran Tao, Zhen Ding, Gan Lu, Jiannan Liu, Fei Xu

**Affiliations:** 1 Department of Non-Communicable Disease Prevention, Jiangsu Provincial Center for Disease Control and Prevention, Nanjing, China; 2 Department of Non-Communicable Disease Prevention, Nanjing Municipal Center for Disease Control and Prevention, Nanjing, China; 3 Department of Respiratory Medicine, Geriatric Hospital of Nanjing Medical University, 30 Luojia Road, Nanjing, 210024 China; 4 Department of Respiratory and Critical Care Medicine, The First Affiliated Hospital, Nanjing Medical University, Nanjing, 210029 Jiangsu China; 5 Department of Environmental Disease Control, Jiangsu Provincial Center for Disease Control and Prevention, Nanjing, China; 6 Nanjing Municipal Center for Disease Control and Prevention, 3, Zizhulin, Nanjing, 210037 China

**Keywords:** COPD, Cigarette smoking, PM_2.5_, Adults

## Abstract

**Background:**

Cigarette smoking and PM_2.5_ are important risk factors of Chronic Obstructive Pulmonary Disease (COPD). However, the joint association of cigarette smoking and PM_2.5_ with COPD is unknown.

**Methods:**

A community-based study was conducted among urban and rural adults aged 40 + years between May and December of 2015 in Jiangsu Province, China. The outcome variable was spirometry-defined COPD. Explanatory measures were smoking status (non-smokers or smokers) and PM_2.5_ exposure [low level (< 75 μg/m^3^) or high level (≥ 75 μg/m^3^)]. Mixed-effects logistic regression models were applied to calculate the odds ratio (OR) and 95% confidence interval (CI) to investigate the associations of cigarette smoking and PM_2.5_ with COPD.

**Results:**

The prevalence of COPD was 11.9% (95% CI = 10.9%, 13.0%) within the overall 3407 participants in this study. After adjustment for potential confounders and community-level clustering effect, smokers tended to develop COPD relative to non-smokers (OR = 2.46, 95% CI 1.76, 3.43), while only smokers exposed to high level PM_2.5_ were more likely to experience COPD (OR = 1.36; 95% CI 1.01, 1.83) compared to their counterparts exposed to low level PM_2.5_. Meanwhile, compared to non-smokers who exposed to low level PM_2.5_, non-smokers who exposed to high level PM_2.5_ (OR = 1.10, 95% CI 0.74, 1.64), smokers who exposed to low (OR = 2.22, 95% CI 1.51, 3.27) or high level PM_2.5_ (OR = 3.14, 95% CI 2.15, 4.59) were, respectively, more like to develop COPD.

**Conclusions:**

Cigarette smoking was positively associated with COPD among overall participants, while PM_2.5_ was in positive relation to COPD among smokers only. Moreover, cigarette smoking and PM_2.5_ might have an additive effect on the risk of COPD among adult smokers aged 40 years or older in China.

## Background

Chronic obstructive pulmonary disease (COPD) is a global public health problem, and the present prevalence of COPD was 11.7–15.8% worldwide [1–3]. For China, the most populous country in the world, COPD has also caused heavy disease burden. The recently estimated prevalence of COPD, defined according to spirometry, was 8.6% among overall adults in China, and this figure was even as high as 13.7% in those aged 40 + years [4]. Moreover, COPD might account for 697.63 million years lived with disability and nearly one million deaths every year in China [4, 5]. Thus, it is a public health priority to reduce the disease burden caused by COPD through population-based intervention programs in China.

Identifying specific modifiable risk factors is critically important for tailored COPD prevention at population level. Cigarette smoking and air pollution are two of such risk factors of COPD [6]. Cigarette smoking has been examined to be significantly associated with COPD either measured as smoking versus non-smoking or assessed based on the number of cigarettes smoked [4, 7, 8]. And it was further documented that cigarette smoking could account for 80–90% of COPD cases [8, 9]. For another modifiable risk factor of COPD, outdoor air pollution was commonly indicated with concentration of particulate matter with a diameter less than 2.5 μm (PM_2.5_) [2, 4]. Moreover, a cutoff of 75 μg/m^3^ was usually employed to classify PM_2.5_ concentration as “low” versus “high” level in population-based studies regarding association of air pollution (indicated with PM_2.5_) with COPD, showing that PM_2.5_ concentration was in significantly positive relation to COPD [4, 10].

The individual link between cigarette smoking, PM_2.5_ and COPD has been investigated, but the joint association of cigarette smoking and PM_2.5_ with COPD was not explored yet. Identifying the potential joint association would be of help for developing risk factor-specific intervention strategies against COPD. To bridge this gap, we conducted a population-based study to examine the combined association of cigarette smoking and PM_2.5_ with COPD among adults in regional China, with a hypothesis that cigarette smoking and PM_2.5_ might exert additive effect on COPD.

## Methods

### Study design and participants

This study was a cross-sectional survey, conducted between May and December of 2015 in Jiangsu province in the eastern region of China [5]. According to the present 5-level administrative strata in China (Central, provincial, municipal/city, district/country, and street/township), Jiangsu province has 13 administrative municipalities/cities. For periodically monitoring mortality, China has established a national disease surveillance point (DSP) system for many years [11]. Recently this mortality DSP system has been integrated with the existing disease prevalence and risk behaviors surveillance system into a new DSP system in China [11]. This new DSP system totally consists of 605 district/county-level DSPs in China [11], including six (three urban districts and three rural counties, each from one municipality/city) from Jiangsu Province.

Eligible participants were household residents aged 40 + years and had been registered for at least 6 months within selected neighborhoods/villages. However, those adults were excluded, if they had cognitive/literal/mental problems, diagnosed cancers, and/or paraplegia. And pregnant women were also not included in the study. The sample size was estimated based on: (1) study design and sampling approach; (2) the odds ratio (OR) presently available for the separate association between cigarette smoking (OR = 1.87), PM_2.5_ (OR = 1.64) and COPD in China [4, 5, 12]; (3) an assumption that an additive effect would exist for combined association of cigarette smoking and PM_2.5_ with COPD; and (4) an expected response rate of 90%. Thus, we determined that approximately 3600 participants would be statistically sufficient for this study.

**Fig. 1:**
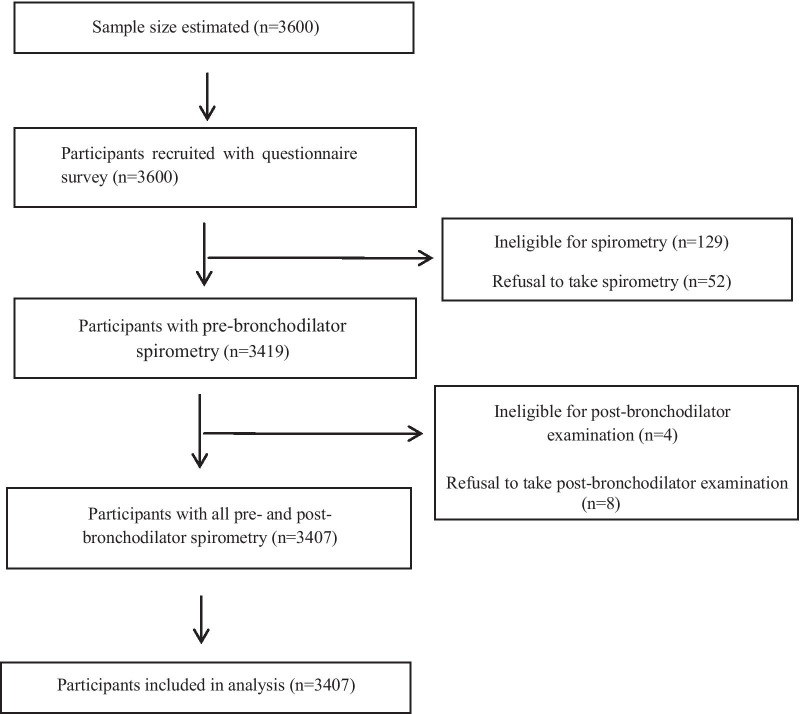
Participants selection flow-diagram

For selection of participants, a multistage sampling method was employed in our study. Firstly, we randomly chose three administrative streets/towns from each of the six provincial DSP districts/counties. Then, we randomly selected two neighborhoods/villages from each chosen street/town. Next, 100 households were randomly determined within each selected neighborhood/village. Finally, one household member was identified as the eligible participant using a KISH grid sampling approach. The participants selection flowchart was shown in Fig. 1.

The study protocol was reviewed and approved by the ethics committee of National Center for Chronic Disease Prevention and Control of China in accordance with the Declaration of Helsinki. Written informed consents were obtained from all participants before the study. All personal identifiable information was deleted prior to data analysis.

### Data collection

A questionnaire survey was conducted via face-to-face interview by our well-trained research staff to gather information on each participant’s socio-demographic characteristics, personal medical history, parental history of respiratory disease, personal respiratory symptoms, and risk factors for respiratory disease (including cigarette smoking). The questionnaire used in this study was a standard one, which was developed and validated by the National Center for Chronic Disease Prevention and Control of China in 2014, and subsequently was used to gather information on COPD in nation/region-level population-based surveys in China in 2015. The relevant data regarding this questionnaire and the nation-wide COPD survey have been published with *Lancet Respiratory Medicine* in 2018 [5]. Participants’ body weight and height were objectively measured to the nearest 0.1 kg (kg) and 0.1 cm (cm), respectively. And body mass index (BMI) was calculated as body weight (kg) divided by the square of body height (m^2^).

### Pulmonary function test

Pulmonary function test referred to pre-bronchodilator and post-bronchodilator spirometry, including forced vital capacity (FVC) and forced expiratory volume in 1 s (FEV1) tests. Each participant’s lung function was tested using a calibrated spirometer (MasterScreen Pneumo, Jaeger, Germany) by certificated staff according to the American Thoracic Society’ recommendations [13, 14]. All participants received pre-bronchodilator spirometry. Subsequently those subjects would receive post-bronchodilator lung function tests 15 min later after 400 μg salbutamol (Ventolin; GlaxoSmithKline, Middlesex, UK) administered, if they were not allergic to salbutamol and with resting heart rate less than 100 bpm.

### Study variables

#### Outcome variable

The outcome variable was COPD, which was diagnosed based on the Global Initiative for Chronic Obstructive Lung Disease 2017 (GOLD 2017) [13]. In China, an individual would be diagnosed as a COPD patient by hospital-based registered physicians, only if he/she received spirometry showing a post-bronchodilator FEV1/FVC < 70% and experience appropriate respiratory symptoms. In the present study, a participant would be diagnosed as having COPD, if he/she: (1) has been diagnosed as a COPD patient by hospital-based registered physicians; or (2) had a post-bronchodilator FEV1/FVC < 70% and appropriate respiratory symptoms [13]. However, for participants with the post-bronchodilator FEV1/FVC < 70%, they would not be diagnosed as COPD patients if their lung function impairment was caused by lung surgery and/or musculoskeletal diseases [13].

### Explanatory measures

The first explanatory measure was cigarette smoking, a main risk factor for COPD. Current smokers were defined as people who smoked ≥ 1 cigarette per day continuously for ≥ 1 year or totally smoked ≥ 18 packs each year, while former smokers referred to those who previously smoked but subsequently gave up smoking for more than 1 year [7]. For participants who did not meet the criteria for either current or former smokers, they were recorded as never smokers [7]. Subsequently, participants were classified into two sub-groups for analysis: smokers (current/former smokers) or non-smokers (never smokers).

The second explanatory variable was PM_2.5_ concentration. Annual mean PM_2.5_ concentrations in 2015 were computed for each survey community based on daily data from Jiangsu provincial environment monitoring system [15], which was officially established by Jiangsu Provincial Department of Ecology and Environment. This monitoring system automatically recorded PM2.5 concentrations consecutively all the time using β-ray particulate matter monitors (MetOne BAM-1020, Met One Instruments company, USA).

In China, the official recommendation of PM_2.5_ concentrations is not exceeding 75 μg/m^3^ in residents areas based on Ambient Air Quality Standards issued by Ministry of Ecology and Environment of China [16]. And, 75 μg/m^3^ was widely accepted as a cutoff of PM_2.5_ concentrations to categorize participants for analysis in previous studies regarding PM_2.5_ exposure and health conditions in China [4, 10]. Therefore, for analysis we classified study subjects into two categories: exposed to ≥ 75 μg/m^3^ or exposed to < 75 μg/m^3^.

For investigating the joint association of cigarette smoking and PM_2.5_ with COPD, participants were also categorized into four sub-groups: non-smokers who exposed to < 75 μg/m^3^ PM_2.5_ (the lowest risk group, the reference), non-smokers who exposed to ≥ 75 μg/m^3^ PM_2.5_, smokers who exposed to < 75 μg/m^3^ PM_2.5_, or smokers who exposed to ≥ 75 μg/m^3^ PM_2.5_ (the highest risk group).

### Covariates

Some classical covariates were controlled for in the multivariate analysis, including age (40–49, 50–59, 60–69, 70 + years old), gender (men vs. women), residential location (urban vs. rural area), socio-economic status, household biomass use, parental history of respiratory diseases and body weight status (BMI < 24, BMI = 24–27, or BMI = 28 +). All of them were treated as categorical variables in the analysis. In this study, the mean value (± SD) of PM_2.5_ was significantly higher in urban areas than that in rural areas (PM_2.5_ in urban vs. rural areas: 81.82 ± 27.76 μg/m^3^ vs. 61.91 ± 29.33 μg/m^3^*, p* < 0.01). The mean concentrations of PM_2.5_ that participants with 13+ years, 9–13 years and 9− years educational attainment exposed to were, respectively, 67.89 ± 12.80 μg/m^3^, 69.57 ± 16.00 and 72.24 ± 19.22 μg/m^3^ (*p* < 0.01), and the PM_2.5_ mean values that the white- and blue-collar exposed to were 72.43 ± 16.60 μg/m^3^ and 71.38 ± 19.42 μg/m^3^ (*p* = 0.13), respectively.

Socio-economic status was indicated with education and occupation, separately. Educational attainment was grouped into three sub-categories based on schooling years completed: 6− years, 7–12 years or 13 + years, while occupation was classified as blue collar (farmer, factory worker, forestry worker, fisher, salesperson, house-worker and vehicle driver) or white collar (office worker, teacher, doctor, academic researcher and government official) [17].

Indoor air pollution was predicted with biomass fuels (wood, grass, and crop residues) used for household cooking or heating [5]. Participants were categorized into two sub-groups: biomass fuel users or non-biomass fuel users. Positive parental history of respiratory diseases referred to that either of parents has been diagnosed with any of the following respiratory diseases: asthma, chronic bronchitis, emphysema, COPD, pulmonary heart disease, or bronchiectasis. Otherwise, negative parental history of respiratory disease was recorded in the analysis.

### Statistical analysis

We compared differences in prevalence of COPD between participants’ selected characteristics via chi-square tests (categorical data) or t-tests (continuous data). Mixed-effects regression models were employed to estimate odds ratios (ORs) and 95% confidence intervals (95%CIs) for examining individual and joint associations of cigarette smoking and PM_2.5_ with COPD. Two models were introduced: model 1 was a univariate analysis with cigarette smoking, PM_2.5_ or their joint categories as the main effect; model 2 was a multivariate analysis with additional consideration of potential risk factors of COPD including age, gender, residence, educational attainment,occupation household biomass use, body weight status. In these two models, study areas were treated as the random effect. We analyze the data with SPSS 21.0 (IBM Corp, Armonk, NY, USA).

## Results

Initially, 3600 eligible participants were recruited, and 3407 (94.6%) completed both the questionnaire survey and spirometry. No significant differences were examined between respondents and non-respondents in terms of age, gender, education, occupation or body weight status. Table 1 displays selected personal characteristics of participants by smoking status in this study. For overall participants, their mean (standard deviation) age was 57.2 (9.9) years; 41.5% were elders (aged 60+ years); 45.8% were men; and 49.1% resided in urban areas. There was no difference in each of these three main socio-demographic characteristics (age, gender and residential location) between our sample population and the standard population of Jiangsu Province in 2015 [18]. Moreover, 33.5% lived in a area with PM_2.5_ ≥ 75 μg/m^3^; 26.1% were biomass fuel users; 31.9% subjects had positive parental history of chronic respiratory diseases; and 17.9% were obese adults.

**Table 1:** Selected socio-demographic and anthropometric characteristics of participants in this study

	Total, n (%)	Never-smokers, n (%)	Smokers, n (%)^a^
N of participants	3407	2073 (60.8)	1334 (39.2)
Mean age (deviation)	57.2 (9.9)	56.27 (9.8)	58.7 (9.7)
Age (years)			
40–49	863 (25.3)	599 (28.9)	264 (19.8)
50–59	1129 (33.1)	698 (33.7)	431 (32.3)
60–69	1020 (29.9)	566 (27.3)	454 (34.0)
70+	395 (11.6)	210 (10.1)	185 (13.9)
Gender			
Men	1561 (45.8)	334 (16.1)	1227 (92.0)
Women	1846 (54.2)	1739 (83.9)	107 (8.0)
Residence			
Urban	1674 (49.1)	1002 (48.3)	672 (50.4)
Rural	1733 (50.9)	1071 (51.7)	662 (49.6)
Educational attainment (years)			
9−	2780 (81.6)	1702 (82.1)	1078 (80.8)
10–12	510 (15.0)	301 (14.5)	209 (15.7)
13+	117 (3.4)	70 (3.4)	47 (3.5)
Occupation^b^			
Blue collar	2384 (70.0)	1523 (73.5)	861 (64.5)
White collar	1023 (30.0)	550 (26.5)	473 (35.5)
Annual mean PM2.5(μg/m^3^)^c^			
< 75	2267 (66.5)	1420 (68.5)	847 (63.5)
≥ 75	1140 (33.5)	653 (31.5)	487 (36.5)
Biomass use^d^			
No	2518 (73.9)	1558 (75.2)	960 (72.0)
Yes	889 (26.1)	515 (24.8)	374 (28.0)
Parental history of respiratory diseases^e^			
Negative	2321 (68.1)	1402 (67.6)	919 (68.9)
Positive	1086 (31.9)	671 (32.4)	415 (31.1)
Body weight status (BMI)^f^			
24−	1296 (38.0)	783 (37.8)	513 (38.5)
24–27	1501 (44.1)	920 (44.4)	581 (43.6)
28+	610 (17.9)	370 (17.8)	240 (18.0)

^a^Smokers referred to either current (continuously smoked at least one cigarette every day for at least 1 year or totally smoked 18+ packs in a year) or former (met the criteria of current smokers previously but now did not smoke for > 1 year) smokers, while non-smokers means the never-smokers. Non-smokers were those not meeting the current/former smokers’ criteria

^b^Blue collar: including farmer, factory worker, forestry worker, fisher, salesperson, houseworker and vehicle driver; White collar: including office worker, teacher, doctor, academic researcher and government official

^c^Classification of PM2.5 was based on “ambient air quality standard (GB3095-2010)” issued by China State Department of Environment Protection in 2010

^d^Typically using wood, grass, crop residues or animal waste for household cooking or heating

^e^Positive parental history of respiratory diseases referred to that either of parents has been diagnosed with any of the following respiratory diseases: asthma, chronic bronchitis, emphysema, COPD, pulmonary heart disease, or bronchiectasis

^f^Body weight status was assessed based on BMI cutoffs recommended for Chinese adults

Table 2 shows the prevalence of COPD among participants by smoking and PM_2.5_ exposure status. The COPD prevalence was 11.9% (95% CI 10.9%, 13.0%) among overall participants, while 6.4% (95% CI 5.3%, 7.4%), 20.6% (95% CI 18.4%, 22.8%), 10.9% (95% CI 9.6%, 12.1%), and 14.1% (95% CI 12.1%, 16.1%) for non-smokers, smokers, those living areas with PM_2.5_ < 75 μg/m^3^ and PM_2.5_ ≥ 75 μg/m^3^, respectively. The prevalence of COPD tended to be higher among men, household biomass fuel users, and those had positive parental history of chronic respiratory diseases. Moreover, COPD prevalence became higher as participants aged, but became lower with their educational attainment or BMI increased. There was no difference in COPD prevalence between urban and rural subjects, or blue and white collars.

**Table 2:** Prevalence of COPD among participants by smoking status and PM2.5 concentration in this study

	Prevalence of COPD														
	Overall (N = 3047)			Smoking status^a^						Annual mean PM_2.5_ concentration^b^ (μg/m^3^)					
				Non-smokers (N = 2073)			Smokers (N = 1334)			< 75 (N = 2267)			≥ 75 (N = 1140)		
	n	% (95%CI)	*P* value	n	% (95%CI)	*P* value	n	% (95%CI)	*P* value	n	% (95%CI)	*P* value	n	% (95%CI)	*P* value
Overall	407	11.9 (10.9, 13.0)		132	6.4 (5.3, 7.4)		275	20.6 (18.4, 22.8)		246	10.9 (9.6, 12.1)		161	14.1 (12.1, 16.1)	
Gender			< 0.001			0.017			0.792			< 0.001			< 0.001
Men	285	18.3 (16.3, 20.2)		31	9.3 (6.2, 12.4)		254	20.7 (18.4, 23.0)		177	16.8 (14.6, 19.1)		108	21.2 (17.6, 24.7)	
Women	122	6.6 (5.5, 7.7)		101	5.8 (4.7, 6.9)		21	19.6 (12.1, 27.2)		69	5.7 (4.4, 7.0)		53	8.4 (6.2, 10.6)	
Age (years)			< 0.001			< 0.001			< 0.001			< 0.001			< 0.001
40–49	28	3.2 (2.1, 4.4)		14	2.3 (1.1, 3.5)		14	5.3 (2.6, 8.0)		25	4.2 (2.6, 5.8)		3	1.1 (0, 2.4)	
50–59	89	7.9 (6.3, 9.5)		29	4.2 (2.7, 5.6)		60	13.9 (10.7, 17.2)		60	7.8 (5.9, 9.7)		29	8.0 (5.2, 10.8)	
60–69	175	17.2 (14.8, 19.5)		49	8.7 (6.3, 11.0)		126	27.8 (23.6, 31.9)		99	14.9 (12.2, 17.6)		76	21.5 (17.2, 25.7)	
70+	115	29.1 (24.6, 33.6)		40	19 (13.7, 24.4)		75	40.5 (33.5, 47.6)		62	25.7 (20.2, 31.2)		53	34.4 (26.9, 41.9)	
Residence			0.112			0.885			0.068			0.530			
Urban	215	12.8 (11.2, 14.4)		63	6.3 (4.8, 7.8)		152	22.6 (19.5, 25.8)		54	10.1 (7.6, 12.7)		161	14.1 (12.1, 16.1)	
Rural	192	11.1 (9.6, 12.6)		69	6.4 (5.0, 7.9)		123	18.6 (15.6, 21.5)		192	11.1 (9.6, 12.6)		0		
Educational attainment (years)			< 0.001			0.069			< 0.001			0.418			< 0.001
9−	356	12.8 (11.6, 14.0)		116	6.8 (5.6, 8.0)		240	22.3 (19.8, 24.7)		210	11.1 (9.7, 12.6)		146	16.3 (13.9, 18.8)	
10–12	43	8.4 (6.0, 10.8)		13	4.3 (2.0, 6.6)		30	14.4 (9.6, 19.1)		32	10.1 (6.8, 13.4)		11	5.7 (2.4, 9.0)	
13+	8	6.8 (2.3, 11.4)		3	4.3 (0.0, 9.0)		5	10.6 (1.8, 19.5)		4	6.2 (0.3, 12.2)		4	7.5 (0.4, 14.7)	
Occupation^c^			0.628			0.153			0.288			0.353			0.664
Blue collar	289	12.1 (10.8, 13.4)		104	6.8 (5.6, 8.1)		185	21.5 (18.7, 24.2)		192	11.2 (9.7, 12.7)		97	14.5 (11.8, 17.2)	
White collar	118	11.5 (9.6, 13.5)		28	5.1 (3.3, 6.9)		90	19.0 (15.5, 22.6)		54	9.8 (7.3, 12.3)		64	13.6 (10.5, 16.7)	
Biomass use^d^			< 0.001			0.011			0.025			0.036			< 0.001
No	270	10.7 (9.5, 11.9)		87	5.6 (4.4, 6.7)		183	19.1 (16.6, 21.5)		161	10.0 (8.5, 11.4)		109	12.0 (9.9, 14.2)	
Yes	137	15.4 (13.0, 17.8)		45	8.7 (6.3, 11.2)		92	24.6 (20.2, 29.0)		85	13.0 (10.4, 15.6)		52	22.1 (16.8, 27.4)	
Parental history of respiratory diseases^e^			0.012			0.011			0.127			0.078			0.100
Negative	255	11.0 (9.7, 12.3)		76	5.4 (4.2, 6.6)		179	19.5 (16.9, 22.0)		159	10.1 (8.6, 11.6)		96	12.9 (10.5, 15.3)	
Positive	152	14.0 (11.9, 16.1)		56	8.3 (6.3, 10.4)		96	23.1 (19.1, 27.2)		87	12.6 (10.1, 15.1)		65	16.5 (12.8, 20.1)	
Body weight status (BMI)^f^			< 0.001			0.701			< 0.001			0.024			0.002
24−	189	14.6 (12.7, 16.5)		52	6.6 (4.9, 8.4)		137	26.7 (22.9, 30.5)		112	12.7 (10.5, 14.9)		77	18.5 (14.7, 22.2)	
24–27	169	11.3 (9.7, 12.9)		60	6.5 (4.9, 8.1)		109	18.8 (15.6, 21.9)		103	10.4 (8.5, 12.3)		66	12.8 (9.9, 15.7)	
28+	49	8 (5.9, 10.2)		20	5.4 (3.1, 7.7)		29	12.1 (8.0, 16.2)		31	7.7 (5.1, 10.3)		18	8.6 (4.8, 12.4)	

^a^Smokers referred to either current (continuously smoked at least one cigarette every day for at least 1 year or totally smoked 18+ packs in a year) or former (met the criteria of current smokers previously but now did not smoke for > 1 year) smokers, while non-smokers means the never-smokers. Non-smokers were those not meeting the current/former smokers’ criteria

^b^Classification of PM2.5 was based on “ambient air quality standard (GB3095-2010)” issued by China State Department of Environment Protection in 2010

^c^Blue collar: including farmer, factory worker, forestry worker, fisher, salesperson, house-worker and vehicle driver; White collar: including office worker, teacher, doctor, academic researcher and government official

^d^Typically using wood, grass, crop residues or animal waste for cooking or heating

^e^Positive parental history of respiratory diseases referred to that either of parents has been diagnosed with any of the following respiratory diseases: asthma, chronic bronchitis, emphysema, COPD, pulmonary heart disease, or bronchiectasis

^f^Body weight status was assessed based on BMI cutoffs recommended for Chinese adults

Table 3 presents individual and joint associations of cigarette smoking and PM_2.5_ with COPD among participants. Among overall participants, after adjustment for age, gender, residence, education, occupation, biomass use, parental history of respiratory diseases, body weight status, PM_2.5_/cigarette smoking and potential clustering effects at study area level, smokers were at 2.46 times odds to experience COPD than non-smokers (OR = 2.46, 95% CI 1.76, 3.43), while participants living in areas with PM_2.5_ ≥ 75 μg/m^3^ were more likely to develop COPD relative to their counterparts living in areas with PM_2.5_ < 75 μg/m^3^ (OR = 1.29, 95% CI 1.02, 1.64). Furthermore, with control for potential confounding factors, compared to non-smokers who exposed to < 75 μg/m^3^ PM_2.5_, non-smokers who exposed to ≥ 75 μg/m^3^ PM_2.5_ (OR = 1.10, 95% CI 0.74, 1.64), smokers who exposed to < 75 μg/m^3^ PM_2.5_ (OR = 2.22, 95% CI 1.51, 3.27) and ≥ 75 μg/m^3^ PM_2.5_ (OR = 3.14, 95% CI 2.15, 4.59) were, respectively, more likely to develop COPD. Such an association was non-significant between non-smoker who exposed to < 75 μg/m^3^ PM_2.5_ and ≥ 75 μg/m^3^ PM_2.5_. However, for those smokers who exposed to ≥ 75 μg/m^3^ PM_2.5_ were at 1.36 (95% CI 1.01, 1.83) times likelihood to experience COPD relative to their counterparts who exposed to < 75 μg/m^3^ PM_2.5_.

**Table 3:** Individual and joint association of smoking and PM2.5 with COPD among participants in this study

Exposure variables		Prevalence of COPD among participants,% (n/N)	OR (95%CI)			
Smoking exposure	Annual mean PM_2.5_ exposure		Model 1^a^		Model 2^b^	
Non-smokers	N/A	6.4 (132/2073)	1		1	
Smokers	N/A	20.6 (275/1334)	2.64 (1.89, 3.68)		2.46 (1.76, 3.43)	
N/A	< 75 μg/m^3^	10.9 (/2,462,267)	1		1	
N/A	≥ 75 μg/m^3^	14.1 (161/1140)	1.26 (1.01, 1.58)		1.29 (1.02, 1.64)	
Non-smokers	< 75 μg/m^3^	6.3 (90/1420)	1		1	
Non-smokers	≥ 75 μg/m^3^	6.4 (42/653)	1.00 (0.67, 1.46)		1.10 (0.74, 1.64)	
Smokers	< 75 μg/m^3^	18.4 (156/847)	2.29 (1.56, 3.37)	1	2.22 (1.51, 3.27)	1
Smokers	≥ 75 μg/m^3^	24.4 (119/487)	2.96 (2.04, 4.31)	1.43 (1.09, 1.88) ^c^	3.14 (2.15, 4.59)	1.36 (1.01, 1.83)^c^

Smoking exposure: −, Non-smoker; + , smoker

Annual mean PM_2.5_ exposure: −, < 75 μg/m^3^; + , ≥ 75 μg/m^3^

^a^Model 1 was a univariate analysis with smoking exposure or annual mean PM2.5 as main effects and study sites as random effects

^b^Model 2 was a multivariate analysis with adjustment for age, gender, residence, education, occupation, biomass use, parental history of respiratory diseases,body weight status, annual mean PM2.5 exposure/cigarette smoking, in addition to Model 1

^c^The OR predicted the likelihood for smokers who exposed to PM2.5 ≥ 75 μg/m^3^ to experience COPD compared to their counterparts who exposed to PM2.5 < 75 μg/m^3^ in this study

## Discussion

In this population-based study, we mainly aimed to explore the joint association of cigarette smoking and PM_2.5_ (predicting outdoor air pollution) with COPD among representative urban and rural adults in regional China. It was observed that either of cigarette smoking or PM_2.5_ concentration was positively associated with COPD, and, furthermore, they additively exerted positive effect on COPD, after control of potential confounding factors. These findings suggested that cigarette smoking and PM_2.5_-based outdoor air pollution might be jointly used as indicators to identify people at high risk in population-based precision intervention campaigns against COPD in China.

The prevalence of spirometry-defined COPD was 11.9% (95% CI 10.9%, 13.0%) among overall participants in our study, 18.3% (95% CI 16.3%, 20.2%) among men and 6.6% (95% CI 5.5%, 7.7%) among women, which were similar to the national estimates among participants with the same age-group in eastern region of China [4, 5]. It has also been estimated that 36.2–40.2% of smokers self-reported being diagnosed with COPD and the population fraction of COPD attributed to cigarette smoking was 22.2% in China in 2015 [4, 5, 19, 20]. Consistent with findings documented in previous studies [4, 5, 19, 20], 20.6% of smokers were diagnosed as COPD patients, and about 67.6% of COPD patients were smokers in our study. Our study shows that COPD prevalence was 14.1% (95% CI 12.1%, 16.1%) among those who resided in areas with PM_2.5_ ≥ 75 μg/m^3^ in this study, which were higher than the figure (9.7%) estimated in a national survey conducted in the same year [4].

Cigarette smoking was the most well-studied and the most important modifiable risk factor of COPD. The independent and positive relationship was solidly established between cigarette smoking and COPD regardless of that cigarette smoking was assessed with categorical (smokers/ex-smokers or non-smokers) or continuous measure (number of cigarettes smoked) [4, 5, 7, 12]. Moreover, even if quitting smoking in time, a participant would continue to experience decline in lung function for years due to the lag-effect of inflammation caused by smoking [20]. In our study, as expected, cigarette smoking was examined to be significantly associated with COPD. After adjustment for potential confounding factors and community-level clustering effects, smokers had a 2.46 times likelihood to experience COPD relative to non-smokers. Such a likelihood for smokers to develop COPD in our study was greater than that for their counterparts in national survey conducted in the same year where the likelihood was less than 2.0 [4, 5].

Another important modifiable risk factor of COPD was outdoor and indoor air pollution [4, 21–23]. With the rapid economic growth over past decades in China, residents might obtain more and more earning and income, and consequently clean energy and kitchen ventilators might have become easily affordable and been widely used for cooking/heating in households, leading to indoor air quality becoming better and better [24, 25]. Moreover, recent studies reported that domestic fuels used, kitchen ventilation and heating in winter was not in significant relation to COPD in China [12, 26]. Therefore, we had indoor air pollution adjusted for in the analysis and then paid particular attention to the relationship between outdoor air pollution (indicated with PM_2.5_ concentration) and COPD in this study. Participants living in air-polluted areas with PM_2.5_ ≥ 75 μg/m^3^ were at 1.29-folds odds for experiencing COPD compared to those living in areas with PM_2.5_ < 75 μg/m^3^ [4], suggesting that air pollution was also in positive relation to COPD.

Cigarette smoking and outdoor air pollution could separately exert positive influence on COPD, and more interestingly and importantly an additive influence by these two risk factors on COPD was observed in this study. The odds for experiencing COPD was 3.14 for smokers who exposed to ≥ 75 μg/m^3^ PM_2.5_, 2.22 for smokers living areas with PM_2.5_ < 75 μg/m^3^ and 1.10 for non-smokers exposed to ≥ 75 μg/m^3^ PM_2.5_, respectively, relative to non-smokers who exposed to < 75 μg/m^3^ PM_2.5_, suggesting a positively gradient association of cigarette smoking and PM_2.5_ with COPD in the study population. Interestingly and meaningfully, the likelihood for experiencing COPD between those exposed to high and low level of PM_2.5_ was significant for smokers but not for non-smokers. It implied that PM_2.5_ might exert influence on the risk of experiencing COPD for cigarette smokers only. This might be due to the relatively fewer COPD patients identified among non-smokers or some unknown underlying mechanisms. Well designed studies with sufficient sample size are in need to explore the PM2.5-COPD association among non-smokers in future.

With respect to the potential mechanisms behind this scenario, there are at least two main explanations. The first is that either cigarette smoking or PM_2.5_ could stimulate some key oxidative and pro-inflammatory molecules in the process of COPD [22, 27, 28]. The second is that both of these two factors could also increase participants’ susceptibility to bacterial and/or viral infections [22, 29, 30]. Therefore, for cigarette smokers exposed to high level of PM_2.5_, they would be more vulnerable to COPD due to such double impact exerted by smoking and PM_2.5_.

This study has several strengths. First, all the COPD patients were identified using spirometry, an objective lung function assessment. Second, outdoor air pollution was also objectively assessed with PM_2.5_ concentration. Third, participants were from urban and rural areas with representativeness of overall population. Fourth, classical confounding factors and clustering effects at study area level were controlled for in the analysis. Fifth, individual and joint association of cigarette smoking and PM_2.5_ with COPD was separately investigated, showing these two modifiable factors might exert additive influence on COPD. Sixth, although collected in 2015, data used in this study were the most recently available population-based information regarding COPD in Jiangsu Province as well as China. Finally, the interesting findings from this study has important public health implications that both cigarette smoking and outdoor PM_2.5_ should be jointly put into consideration for developing population-based precision prevention campaigns to fight COPD.

Regardless of the strengths, this study also has some limitations. First, because of the nature of cross-sectional study, the association examined in this study did not imply any causal direction. Second, due to lack of data, cigarette smoking, one of the two main explanatory variables, could not be used based on the number of cigarettes smoked, and thus the dose–response relationship between cigarettes smoked and CODP was not able to be assessed. Third, as only yearly mean values of PM_2.5_ were available, we could examine the association between PM_2.5_ and COPD using PM_2.5_ as continuous or peak measures in our study. Fourth, the cutoff of PM_2.5_ concentrations used to classify participants was 75 μg/m^3^ in this study. Although this cutoff was officially recommended by Ministry of Ecology and Environment of China, it was much higher than that recommended by the WHO or the U.S. Environmental Protection Agency. Therefore, when the association between PM_2.5_ and COPD in this study was interpreted, the specific PM_2.5_ cutoff should be put into consideration. Fifth, participants’ personal protective approaches against outdoor air pollution were not considered due to data limitation. Thus, the findings in this study should be interpreted prudently. In future, well-designed large-scale prospective population-based observational or even experimental studies are warranted to further investigate the joint association of cigarette smoking and PM_2.5_ with COPD in China.

## Conclusions

Cigarette smoking and PM_2.5_ were individually in positive relation to COPD, and moreover they might exert additive influence on COPD among urban and rural adult smokers in regional China. This study has important public health significance that population-based precision COPD prevention campaigns should be tailored for specific participants with consideration of multiple risk factors.

## Data Availability

The related data and material will be available upon request to either of the two corresponding authors.

## Abbreviations

COPD: Chronic obstructive pulmonary disease
GOLD: Global Initiative for Chronic Obstructive Lung Disease
FVC: Forced vital capacity
FEV1: Forced expiratory volume in 1 s
PM_2.5_: Particulate matter with a diameter less than 2.5 μm
DSP: Disease surveillance point
OR: Odds ratio
CI: Confidence interval
BMI: Body mass index
